# White Matter Abnormalities in Patients With Parkinson's Disease: A Meta-Analysis of Diffusion Tensor Imaging Using Tract-Based Spatial Statistics

**DOI:** 10.3389/fnagi.2020.610962

**Published:** 2021-01-28

**Authors:** Xia Wei, Chunyan Luo, Qian Li, Na Hu, Yuan Xiao, Nian Liu, Su Lui, Qiyong Gong

**Affiliations:** ^1^Department of Radiology, Functional and Molecular Imaging Key Laboratory of Sichuan Province, Huaxi MR Research Center (HMRRC), West China Hospital, Sichuan University, Chengdu, China; ^2^Psychoradiology Research Unit of the Chinese Academy of Medical Sciences (2018RU011), West China Hospital of Sichuan University, Chengdu, China; ^3^Department of Radiology, National Clinical Research Center for Geriatrics, West China Hospital, Sichuan University, Chengdu, China

**Keywords:** meta-analysis, Parkinson disease, diffusion tensor imaging, tract-based spatial statistics, fractional anisotropy, seed-based d mapping

## Abstract

**Background:** Tract-based spatial statistics (TBSS) studies based on diffusion tensor imaging (DTI) have revealed extensive abnormalities in white matter (WM) fibers of Parkinson's disease (PD); however, the results were inconsistent. Therefore, a meta-analytical approach was used in this study to find the most prominent and replicable WM abnormalities of PD.

**Methods:** Online databases were systematically searched for all TBSS studies comparing fractional anisotropy (FA) between patients with PD and controls. Subsequently, we performed the meta-analysis using a coordinate-based meta-analytic software called seed-based d mapping. Meanwhile, meta-regression was performed to explore the potential correlation between the alteration of FA and the clinical characteristics of PD.

**Results:** Out of a total of 1,701 studies that were identified, 23 studies were included. Thirty datasets, including 915 patients (543 men) with PD and 836 healthy controls (449 men), were included in the current study. FA reduction was identified in the body of the corpus callosum (CC; 245 voxels; *z* = −1.739; *p* < 0.001) and the left inferior fronto-occipital fasciculus (IFOF) 118 voxels; *z* = −1.182; *p* < 0.001). Both CC and IFOF maintained significance in the sensitivity analysis. No increase in FA was identified, but the percentage of male patients with PD was positively associated with the value of FA in the body of the CC.

**Conclusions:** Although some limitations exist, DTI is regarded as a valid way to identify the pathophysiology of PD. It could be more beneficial to integrate DTI parameters with other MRI techniques to explore brain degeneration in PD.

## Introduction

Parkinson's disease (PD) is a neurodegenerative disorder characterized by cardinal symptoms, including tremor, rigidity, bradykinesia, and postural instability (Tolosa et al., 2006). Although PD is defined by its motor symptoms, non-motor symptoms such as depression, sleep disturbance, autonomic dysfunction, and olfactory dysfunction are also present in patients with PD, and may even predate the emergence of the classic motor features (Berg et al., 2013). Over the past generations, the incidence and prevalence rates of PD have increased because of the general aging of the population and other factors (GBD 2016 Parkinson's Disease Collaborators, 2018). The heterogeneous symptoms and the huge burden of PD have prompted scientists to explore the neural basis of this disease to allow for both better diagnosis and intervention.

Numerous studies have sought to explain the mechanism behind PD *via* several different factors, e.g., genetic high risk (Chung et al., 2011; Wile et al., 2017), pathological findings of α-synuclein (Kalia, 2019), and PET with the dopamine tracer (Wile et al., 2017; Kalia, 2019). Nevertheless, these studies mostly focused on the dopamine pathway; however, patients with PD did not present with the symptoms resulting from a dopamine deficit. The development of MRI techniques offers scientists more ways, which are generally less invasive and more cost-effective, to gain insight into the neural basis of PD, bridge the cerebral changes to the clinical symptoms, and predict the progression of disease in the early stage. In numerous MRI studies, patients with PD showed extensive morphological abnormalities. A recent review summarized these abnormalities as (Sarasso et al., 2020) cortical atrophy (e.g., the caudate, the temporal/hippocampal, the frontal areas, and so on) that had accumulated with the progression of the disease and finally reached a plateau. Subsequently, a new question arose: whether these abnormalities were developed independently or resulted from the degeneration of white matter (WM) fibers. There is a hypothesis that the degeneration of WM tracts is the cause of cerebral atrophy and connectivity changes. Some studies (Lee et al., 2011; Duncan et al., 2016; Rektor et al., 2018) have suggested that changes in the WM precede changes in the gray matter (GM) in patients with PD.

Diffusion tensor imaging (DTI) is a newly developed MRI technique that can examine WM properties at a microstructural level (Basser, 1995) by encoding information about the directionality and magnitude of water molecules. Fractional anisotropy (FA), mean diffusivity (MD), axial diffusivity (AD), and radial diffusivity (RD) are four parameters derived from DTI data to assess the WM properties (Andica et al., 2020). In the past, increasing interest in axonal analyses using DTI has deepened our understanding of the WM damage in the pathology of PD. For example, a study demonstrated that neurodegenerative microstructural alterations in patients with PD were different from normal physiological aging (Koirala et al., 2019). However, there is a great deal of inconsistency between the findings of various studies. While some studies (Kim et al., 2013; Carriere et al., 2014; Rossi et al., 2014; Duncan et al., 2016; Acosta-Cabronero et al., 2017; Georgiopoulos et al., 2017; Luo et al., 2017; Mishra et al., 2019) observed no significant differences in FA between patients with PD and healthy controls (HCs), other studies reported decreased FA in various regions, including the corpus callosum (CC; Chen Y. S. et al., 2017); left inferior fronto-occipital fasciculus (IFOF; Chen Y. S. et al., 2017); cingulum (Karagulle Kendi et al., 2008); insular (Chiang et al., 2017); thalamus (Youn et al., 2015); and the parietal, occipital, and cerebellar WM regions (Zhang et al., 2011). There have been studies reporting increased FA in the WM regions of PD, for example, the brainstem (Youn et al., 2015; Lorio et al., 2019), cerebellar, anterior corpus callosal, and inferior fronto-occipital WM (Taylor et al., 2018).

To eliminate the inconsistencies between these different studies, coordinate-based meta-analyses (Albrecht et al., 2019; Suo et al., 2020) on DTI studies were carried out, and decreases in FA were found in the CC, the left middle cerebellar peduncles (MCPs), the left IFOF, and the right inferior longitudinal fasciculus of patients with PD compared with that of HCs. These analyses combined whole-brain tract-based spatial statistics (TBSS; Smith et al., 2006) and voxel-based analyses (VBA) studies, thus attenuating the bias of region-of-interest (ROI) selection. However, VBA is inevitable with partial volume effects and misregistration errors. Compared to VBA, TBSS restricts the analysis to the center of major WM tracts and can more accurately identify WM abnormalities (Rae et al., 2012). To determine the inherent methodical differences between TBSS and VBA, a meta-analysis solely based on TBSS studies was needed.

Therefore, this study carried out a meta-analysis of only TBSS studies to identify the most robust WM microstructural abnormalities in patients with PD. Seed-based d mapping (SDM) software, a voxel-based quantitative meta-analysis approach, was performed in this study. Compared with activation likelihood estimation (ALE), another frequently used meta-analysis software, the advantage of SDM is that it considers null results. In addition, the potential relationship between clinical parameters [age, sex, the Unified Parkinson Disease Rating Scale-III (UPDRS-III) score, disease duration, the Mini-Mental State Examination (MMSE), and the levodopa equivalent daily dose (LEED)] and WM microstructural abnormalities in patients with PD was explored by regression analysis.

## Materials and Methods

### Study Selection

**Figure 1:**
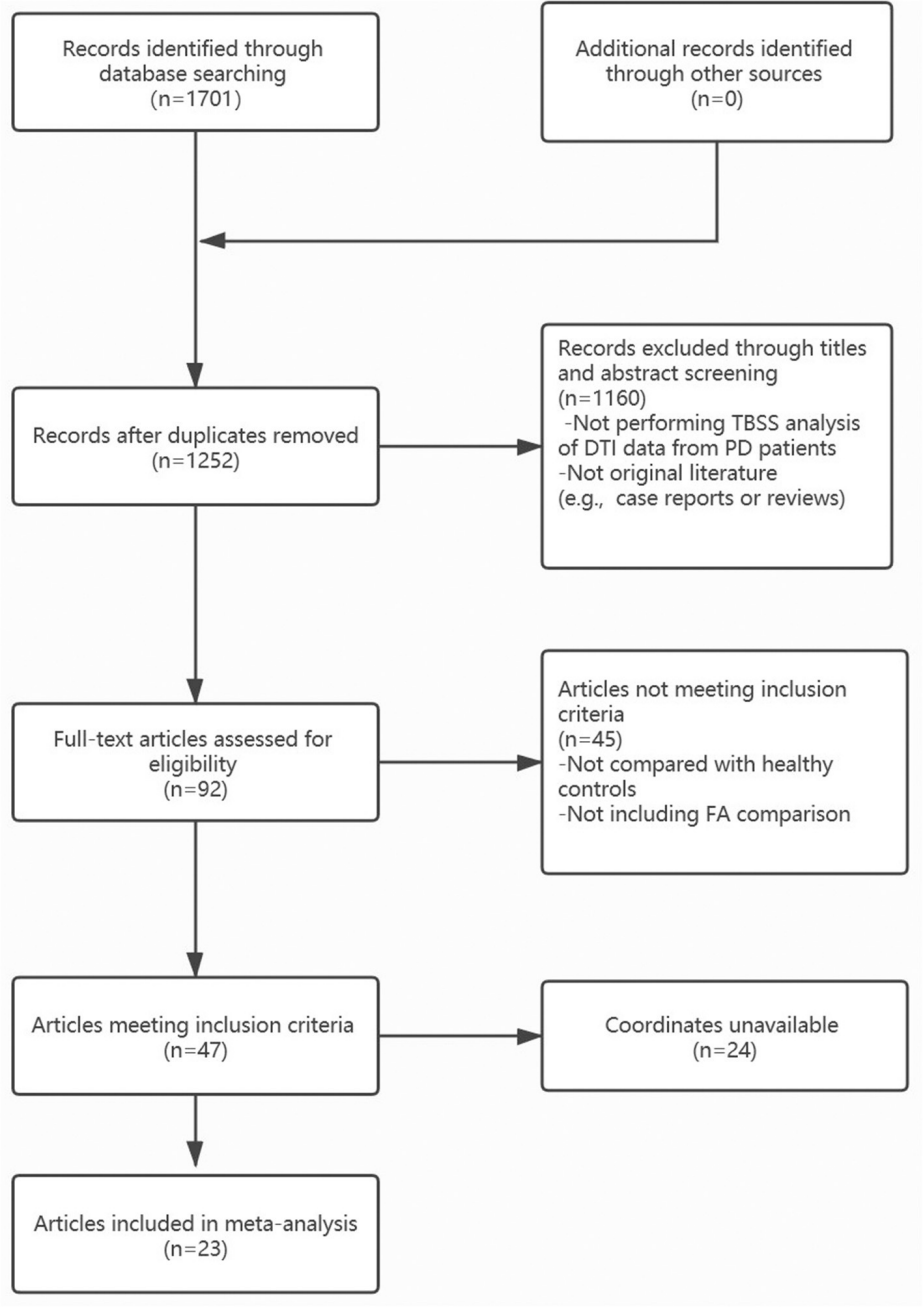
Flow chart for identifying Parkinson's disease (PD) studies.

We searched for pertinent literature in PubMed, Embase, and Web of Science. The following keywords were used to identify relevant articles published up to December 2020: “Parkinson” or “Parkinson's disease”; “tract-based spatial statistical;” or “diffusion tensor” or “fractional anisotropy.” The flow diagram of the literature search is presented in Figure 1, and the inclusion/exclusion criteria are explained in the Supplementary Methods. In summary, only peer-reviewed whole-brain TBSS studies comparing WM abnormalities in patients with PD to HCs were included in this analysis, even those that report null results. The corresponding authors of studies that met all of the above inclusion criteria, but lacked global brain coordinates, were contacted to obtain additional information. Moreover, studies that used multiple independent patient samples were compared separately with the same HC groups, and the appropriate results were regarded as separate datasets. There were no overlapping samples in all included studies. All included studies were assessed and scored for quality using a 15-point checklist adapted from a previously published meta-analysis (Chen et al., 2016; Supplementary Table 1). A similar search strategy was used to explore underlying consistent MD changes in patients with PD compared to HCs. Details are presented in the Supplementary Methods.

### Data Extraction

One author (CYL) searched the literature, and another author (XW) extracted demographic characteristics (sample size, age, and sex), clinical data (disease duration, UPDRS-III, MMSE, medication status, and LEED), imaging parameters, statistical thresholds, and major findings from the included studies. For studies reported with interquartile range and median, we calculated the mean and SD (Wan et al., 2014; Luo et al., 2018; http://www.math.hkbu.edu.hk/tongt/papers/median2mean.html). The three-dimensional coordinates were extracted according to the anisotropic effect-size-based algorithms AES-SDM methods (Radua et al., 2014). All information was double-checked, and decisions were reached after discussing the disagreements.

### Seed-Based d Mapping Meta-Analysis and Fiber Tracking

We used AES-SDM software (version 5.15) to detect consistent FA abnormalities in patients with PD compared with HCs and used DTI-Query software (Stanford, USA) to exhibit the fibers crossing peak coordinates of FA alteration.

Following the tutorial of SDM (Radua and Mataix-Cols, 2009; Radua et al., 2014), we first recreated statistical maps and effect-sized maps of the coordinates extracted from each study (“FA,” anisotropy = 1, isotropic full width at half maximum FWHM = 20 mm, mask = “TBSS,” 10 randomizations). Then, individual study maps were entered into the meta-analysis, and the outcome was further tested in terms of sensitivity and heterogeneity, including jackknife sensitivity, heterogeneity, and publication bias analysis. The analytical parameters applied in the present study were as follows: voxel threshold *p* = 0.005, peak height threshold *Z* = 1.00, and cluster size threshold = 100 voxels, and the outcome maps were visualized using MRIcron software (University of South Carolina, USA). Subgroup analysis of studies reported with corrected results, studies with 3T scanner, and studies with a b-value of 1,000 s/mm^2^ were carried out. Finally, meta-regressions using a linear model were performed to detect the relationship between WM abnormalities and clinical parameters (age, sex, disease duration, UPDRS-III, MMSE, and LEED). We selected a more conservative threshold of *p* = 0.0005, as used in previous studies (Chen et al., 2016; Barona et al., 2019; Li et al., 2020) with peak height threshold *Z* = 1.00, and cluster size threshold = 10 voxels. With DTI-Query software used in previous studies (Li et al., 2012; Zhang et al., 2018), we tracked the probable fibers across the peak coordinates detected by SDM. More details of the SDM method and DTI-Query are presented in the Supplementary Methods.

## Results

### Included Studies and Sample Characteristics

A total of 23 whole-brain TBSS studies (Hattori et al., 2012; Kamagata et al., 2013; Kim et al., 2013; Melzer et al., 2013; Agosta et al., 2014; Carriere et al., 2014; Worker et al., 2014; Diez-Cirarda et al., 2015; Ji et al., 2015; Vercruysse et al., 2015; Vervoort et al., 2016; Wen et al., 2016; Acosta-Cabronero et al., 2017; Chen B. et al., 2017; Georgiopoulos et al., 2017; Luo et al., 2017; Firbank et al., 2018; Li et al., 2018; Minett et al., 2018; Guan et al., 2019; Quattrone et al., 2019; Inguanzo et al., 2020; Pelizzari et al., 2020) were identified using our search protocol. Six of them compared separate independent patient subgroups with the same HC groups, with details present in Table 1. Thus, a total of 30 datasets were included in our meta-analysis. Eight datasets revealed decreased FA in patients with PD in distributed regions, involving commissural fibers (e.g., CC), association fibers [e.g., IFOF and uncinate fasciculi (UF)], and projection fibers [e.g., middle cerebellar peduncle (MCP), interior capsule (IC), and anterior corona radiata (ACR)]. In addition, 22 datasets found no FA alteration in patients with PD compared to HCs. A total of 915 patients with PD (543 men, mean age 64.89 years, mean disease duration 5.65 years, mean UPDRS-III 26.83 years) and 836 matched HCs (mean age 65.19 years, males 449) were analyzed. All patients were diagnosed with PD, excluding any other Parkinsonism syndromes. The field strength of most of these studies was 3T (26/30 datasets), and the b-value was mostly 1,000 s/mm^2^ (21/30 datasets). The details of the demographic and clinical characteristics and study information of all included FA studies are presented in Table 1.

**Table 1:** Demographic and clinical characteristics of participants in the 23 Parkinson's disease studies (30 datasets) included in the meta-analysis.

**Study (subgroup)**	**PD**							**HC**		**Study information**					
	**Participants (male)**	**Age, yrs**	**Disease duration**	**UPDRS-III**	**MMSE**	**Medication status**	**LEDD, mg/day**	**Participants (male)**	**Age, yrs**	**Scanner**	**Direction**	***b*-value**	**Software**	**Threshold**	**Major findings**
(Pelizzari et al., 2020) (LPD)	9 (4)	65.99	3.81	17	NA	On-state	158.85	17 (9)	63.16	1.5	64	1,500	FSL	*P* < 0.05 (FWE)	No significant FA alteration
(Pelizzari et al., 2020) (RPD)	12 (7)	68.15	2.18	18.17	NA	On-state	269.06	17 (9)	63.16	1.5	64	1,500	FSL	*P* < 0.05 (FWE)	No significant FA alteration
(Inguanzo et al., 2020; Pelizzari et al., 2020) (PD1)	15 (13)	NA	NA	NA	NA	On-state	NA	33 (18)	NA	3	30	1,000	FSL	P < 0.05 (FWE)	left IFOF
(Inguanzo et al., 2020) (PD2)	21 (14)	NA	NA	NA	NA	On-state	NA	33 (18)	NA	3	30	1,000	FSL	*P* < 0.05 (FWE)	No significant FA alteration
(Inguanzo et al., 2020) (PD3)	26 (19)	NA	NA	NA	NA	On-state	NA	33 (18)	NA	3	30	1,000	FSL	*P* < 0.05 (FWE)	No significant FA alteration
(Quattrone et al., 2019)	37 (26)	72	7.8	33.26	21.87	Off-state	NA	38 (15)	72.6	3	27	1,000	FSL	*P* < 0.05 (FWE)	No significant FA alteration
(Guan et al., 2019)	65 (32)	55.5	4.7	27.1	27.8	Off-state	NA	46 (21)	57.8	3	15	1,000	FSL	*p* < 0.001, cluster-based Corr	right UF
(Li et al., 2018)	31 (16)	60.5	NA	26.4	25.26	NA	NA	22 (12)	59.7	3	32	1,200	FSL	*P* < 0.05 (FWE)	bilateral CI, CE, ACR, SL, genu, body, and pad of CC
(Firbank et al., 2018) (PD-nonVH)	19 (17)	72.3	NA	34.7	25.6	NA	673.5	20 (14)	75.4	3	64	1,000	FSL	*P* < 0.05 (TFCE)	No significant FA alteration
(Firbank et al., 2018) (PD-VH)	17 (13)	75.5	NA	55.9	23.1	NA	717.3	20 (14)	75.4	3	64	1,000	FSL	P < 0.05 (TFCE)	wide spread FA reduction
(Minett et al., 2018)	93 (61)	64.3	0.53	25.9	NA	NA	161	48 (28)	66	3	64	1,000	FSL	*P* < 0.05 (TFCE_FWE)	No significant FA alteration
(Luo et al., 2017) (TD PD)	30 (16)	53.42	2	25.37	NA	Off-state	262	26 (13)	54.46	3	25	1,000	FSL	*P* < 0.05 (FWE)	No significant FA alteration
(Luo et al., 2017) (NoTD PD)	30 (15)	52.55	2.35	22.27	NA	Off-state	305	26 (13)	54.46	3	25	1,000	FSL	*P* < 0.05 (FWE)	No significant FA alteration
(Chen B. et al., 2017)	18 (7)	62.28	3.06	17.39	NA	Off-state	NA	24 (11)	62.88	3	25	1,000	FSL	*P* < 0.017, TFCE & Bonferroni Corr	left hippocampus, body of CC
(Acosta-Cabronero et al., 2017)	25 (20)	63.6	6	16.3	26.7	On-state	748	50 (28)	63.6	3	30	1,000	FSL	*P* < 0.001, Uncorr	No significant FA alteration
(Georgiopoulos et al., 2017)	22 (12)	68.35	7	21.08	29.36	On-state	NA	13 (5)	67.64	3	32	800	FSL	*P* < 0.05 (TFCE)	No significant FA alteration
(Vervoort et al., 2016) (TD PD)	16 (9)	55.1	4.8	28.9	28.9	Off-state	249.2	19 (14)	58.1	3	61	1,300	FSL	*P* < 0.05 (TFCE)	No significant FA alteration
(Wen et al., 2016)	87 (55)	62.01	0.63	25.14	NA	Drug-naïve	0	60 (40)	60.33	3	64	1,000	FSL	*P* < 0.01 (TFCE)	No significant FA alteration
(Vercruysse et al., 2015) (PD with FOG)	11 (8)	68.6	9.5	36.6	27.2	On-state	703.8	15 (11)	68.1	3	25/40/75	700/1,000/2,800	FSL	*P* < 0.05 (FDR)	right MCP
(Vercruysse et al., 2015) (PD without FOG)	15 (11)	67.6	7.6	32.5	28.3	On-state	461.3	15 (11)	68.1	3	25/40/75	700/1,000/2,800	FSL	*P* < 0.05 (FDR)	No significant FA alteration
(Ji et al., 2015)	20 (11)	64.2	4.64	28.76	NA	Off-state	NA	20 (10)	59.95	3	30	1,000	FSL	*P* < 0.05 (TFCE_FWE)	Body of CC
(Diez-Cirarda et al., 2015)	37 (22)	67.97	6.96	21.72	NA	On-state	808.59	15 (11)	65.07	3	32	1,000	FSL	*p* < 0.001 uncorrected.	right UF
(Worker et al., 2014)	17 (9)	63.9	6.6	21.8	29.5	On-state	NA	17 (9)	63.9	3	64	1,300	FSL	*P* < 0.017, TFCE & Bonferroni Corr	No significant FA alteration
(Carriere et al., 2014) (Apathetic PD)	10 (6)	67.2	11.9	28.1	NA	On-state	857.2	10 (4)	66.8	3	15	1,000	FSL	*P* < 0.05 (TFCE)	No significant FA alteration
(Carriere et al., 2014) (Non-apathetic PD)	10 (6)	60.7	11.9	28.7	NA	On-state	1,072.10	10 (4)	66.8	3	15	1,000	FSL	*P* < 0.05 (TFCE)	No significant FA alteration
(Agosta et al., 2014)	43 (29)	65.8	9.1	32.6	27.6	On-state	607.1	33 (17)	64	1.5	12	1,000	FSL	*P* < 0.05 (FWE)	No significant FA alteration
(Kamagata et al., 2013)	20 (8)	71.6	7.83	NA	25.7	On-state	464.2	20 (10)	72.7	3	32	1,000	FSL	*P* < 0.05 (TFCE_FWE)	No significant FA alteration
(Kim et al., 2013)	64 (22)	62.9	5.3	NA	NA	Off-state	NA	64 (22)	63	3	15	800	FSL	*P* < 0.05 (TFCE_FWE)	No significant FA alteration
(Melzer et al., 2013)	63 (43)	64	3.7	25.3	29	On-state	208	32 (22)	70.1	3	28	1,000	FSL	*P* < 0.05 (TFCE)	No significant FA alteration
(Hattori et al., 2012)	32 (12)	75.9	5.8	20	27.8	NA	NA	40 (18)	76.9	1.5	12	1,000	FSL	*P* < 0.05 (TFCE)	No significant FA alteration
Total	915 (543)	64.89	5.66	26.84	26.92		484.79	836 (449)	65.19						

*PD, Parkinson's Disease; HC, healthy controls; UPDRS-III, Unified Parkinson Disease Rating Scale-III; RPD, right-dominant symptom PD patients; LPD, left-dominant symptom PD patients; PD1= PD1 had lower gray matter (GM) volumes than HCs mainly in occipital and medial temporal; PD2, PD2 had GM atrophy compared with HC mainly in bilateral orbital and prefrontal cortical regions; PD3, PD3 did not show significant GM volume differences compared with HC; VH, visual hallucination; TD, tremor dominated; FOG, freezing of gait; PIGD, postural instability and gait difficulty; NC, normal cognition; H&Y, Hoehn & Yahr staging; NA, not available; MRI, Magnetic Resonance Image; Uncorr= uncorrected; Corr, corrected; FEW, family-wise error; TFCE, threshold-free cluster enhancement; FA, fractional anisotropy; MCP, middle cerebellar peduncle; UF, uncinate fasciculus; CC, corpus callosum; CI, capsula interna; CE, capsula externa; ACR, anterior corona radiata; SL, sagittal layer*.

### Pooled Voxel-Based Meta-Analysis

**Figure 2:**
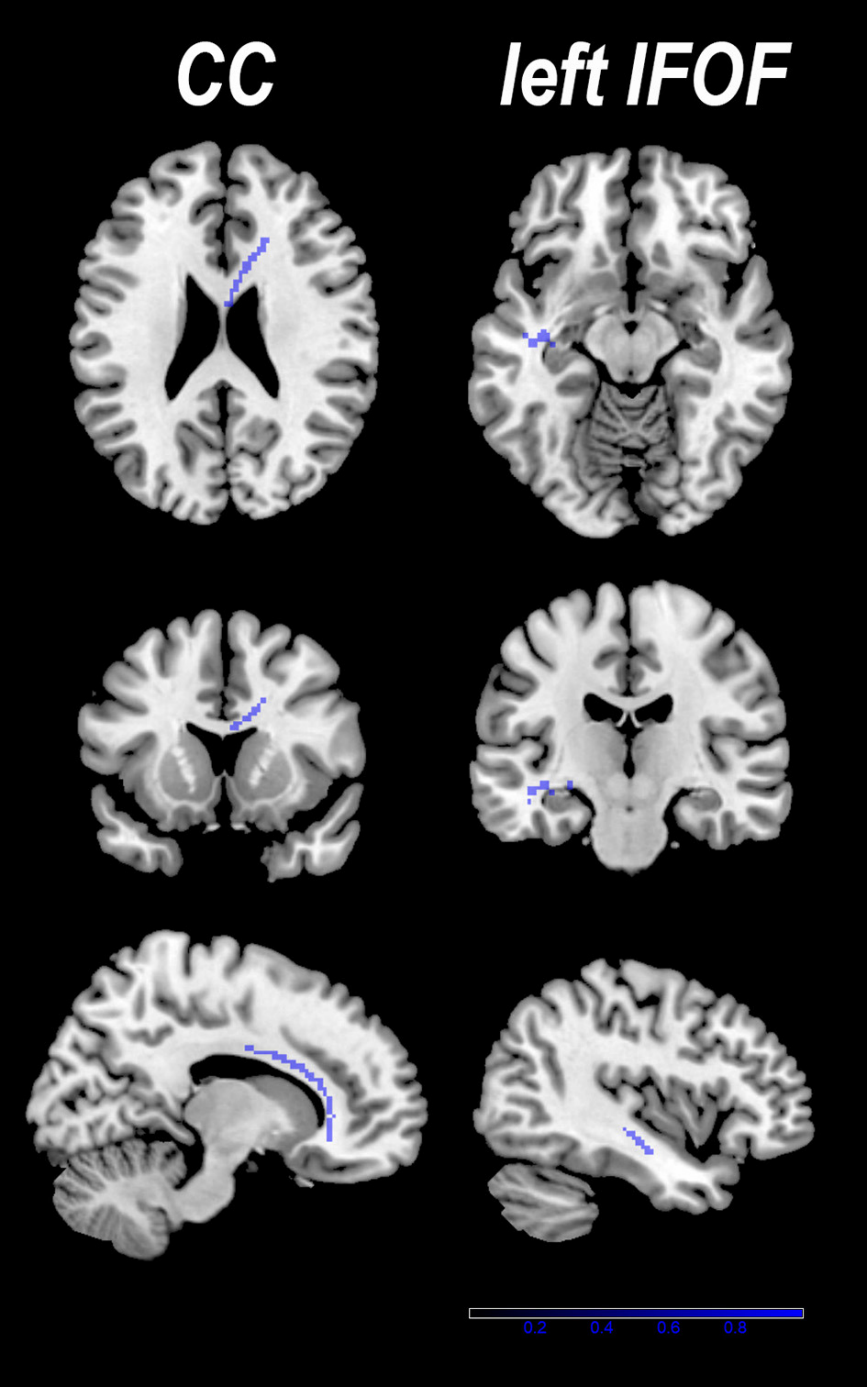
Two clusters with decreased fractional anisotropy (FA) were identified in patients with PD compared with healthy controls (HCs). Results are overlaid on the axial sections of the Montreal Neurological Institute standard brain in neurological convention (right is right).

**Figure 3:**
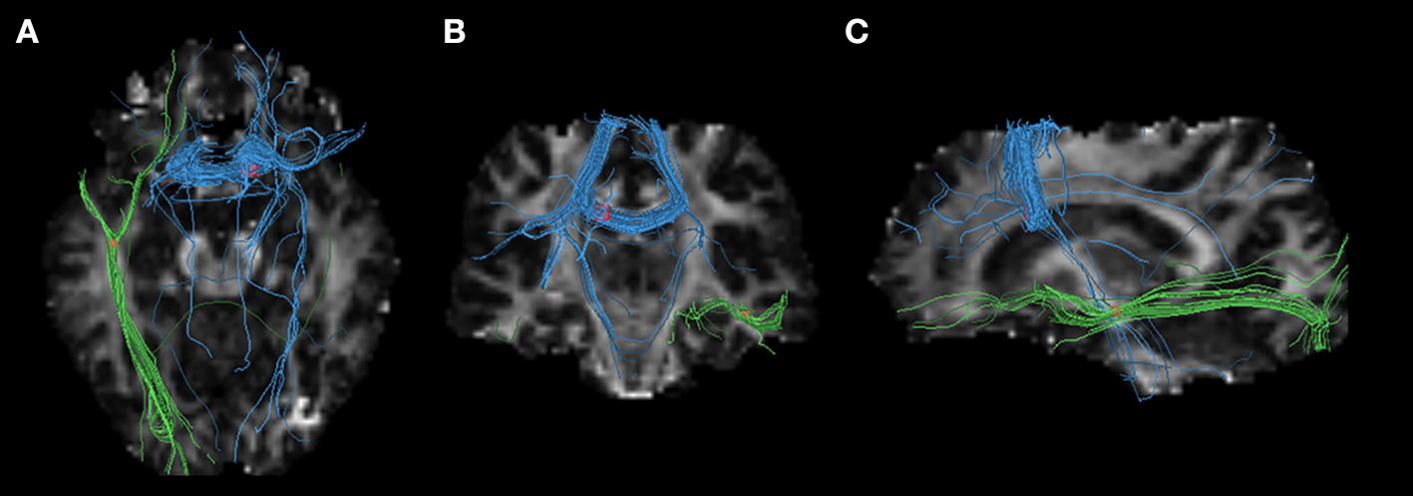
Three-dimensional images showing white matter tracts traversing two bounding boxes centered at were separately mapped with DTI-Query in a single normal individual. The left image **(A)** observed from above, the middle image **(B)** observed from the front, and the left side image **(C)** observed from the left of the brain. Tracts include the interhemispheric fibers running through the body of the CC (blue) connecting the interhemispheric MA and supplementary motor areas (SMAs), and the left inferior fronto-occipital fasciculus (IFOF) crossing left temporal lobe. Axial, coronal, and sagittal slices mapping the FA values are shown in the background for illustrative purposes.

The pooled meta-analysis of 30 datasets identified a decrease of FA in two clusters of patients with PD compared to HCs (Table 2 and Figure 2). The first cluster (245 voxels; *z* = −1.739; *p* < 0.001) peaked at the body of the CC (Montreal Neurological Institute (MNI) space: 12, 14, and 24), extending to the genu of CC. This is because the CC is interhemispheric, even if it is represented in the right hemisphere. The second cluster (118 voxels; *z* = −1.182; *p* < 0.001) included the sagittal stratum, the fornix, and the stria terminalis of the left hemisphere, peaked at the left IFOF (Montreal Neurological Institute (MNI) Space: −42, −18, −12). The main fibers likely to pass through the above peak coordinates are shown in Figure 3. No increase in FA was identified.

**Table 2:** Regional differences in FA between patients with PD and healthy controls.

**WM Tract**	**Voxels**	**MNI Coordinates**			**SDM Z score**	**P, uncorrected**	**Cluster breakdown (voxels)**	**Jackknife**
		**X**	**Y**	**Z**				
Corpus callosum	245	12	14	24	−1.739	0.000004888	Corpus callosum (242)	30/30
Left inferior network, inferior fronto-occipital fasciculus	118	−42	−18	−12	−1.182	0.000615716	Left inferior network, inferior fronto-occipital fasciculus (44) Corpus callosum (19) Left cortico-spinal projections (12) Left optic radiations (12) Left pons (12) Left inferior network, inferior longitudinal fasciculus (10)	27/30

* *< 10 voxels are not represented in the breakdown of voxels*.

*PD, Parkinson's Disease; FA, fractional anisotropy; WM, white matter; MNI, Montreal Neurological Institute Space; SDM, Seed-based d Mapping; Jackknife: The jackknife sensitivity analysis column gives the number of studies whose omission does not affect the finding*.

### Reliability Analysis

None of the clusters with altered FA showed statistically significant between-group heterogeneity or publication biases (Supplementary Figure 1). All clusters are highly reliable in jackknife sensitivity analysis (Supplementary Table 2), especially the body of CC, by remaining significant in all iterations. This indicates that all clusters are highly reliable.

Subgroup analysis findings of studies with corrected results and studies with 3T scanner reproduced pooled results. However, subgroup analysis of studies with a b-value of 1,000 s/mm^2^ failed to find any FA difference between patients with PD and HCs (Supplementary Table 3).

### Result of Meta-Regression Analysis

**Figure 4:**
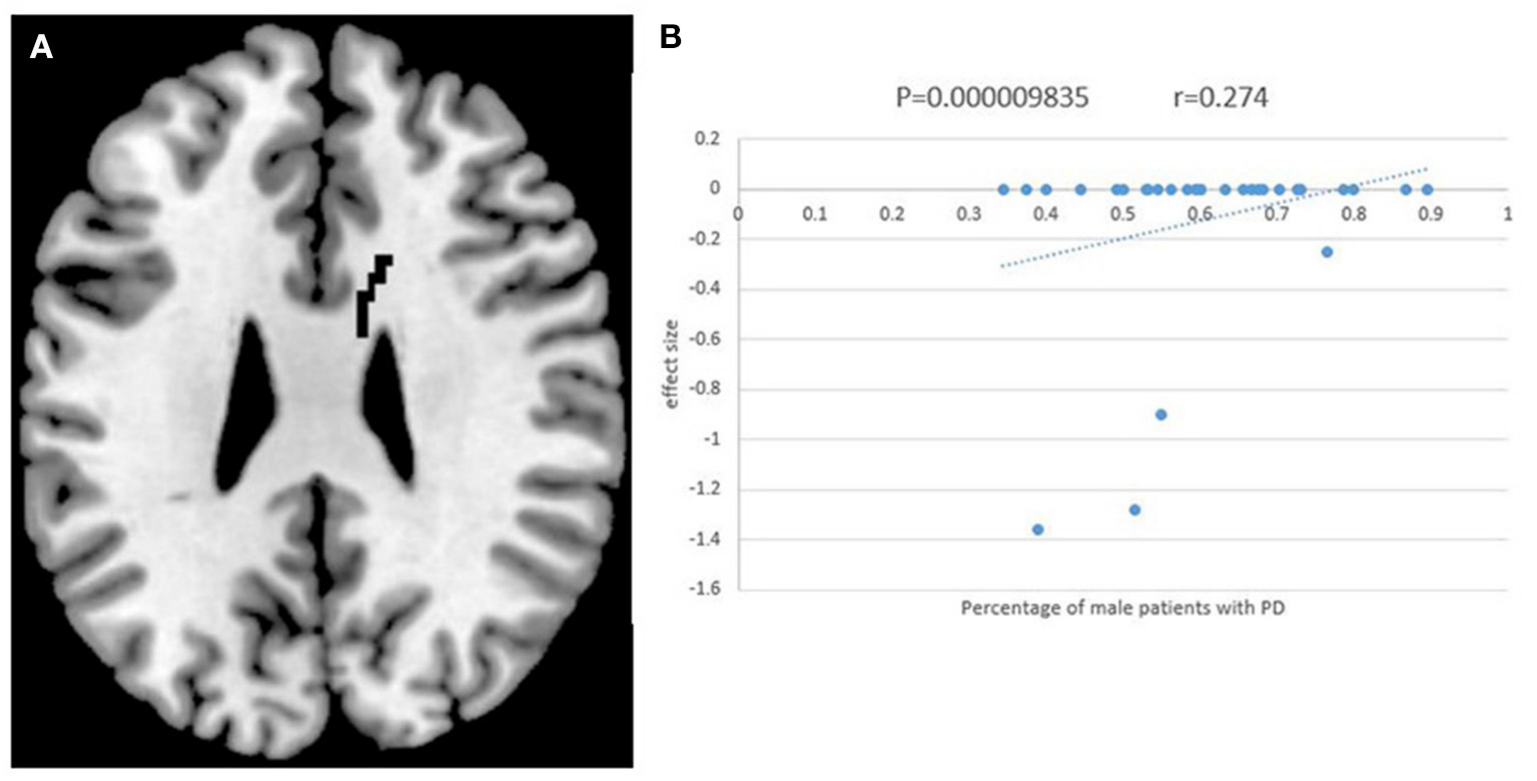
The results of meta-regression analysis. The percentage of male patients with PD was positively correlated with FA in the body of corpus callosum (CC). The effect sizes to create this plot were extracted from the peak of the maximum slope difference, and each study is represented as a dot. The regression line (meta-regression signed differential mapping slope) is shown.

The percentage of male patients with PD was positively associated with the FA value in the body of the CC (Figure 4). Other information on the clinical variables, including the mean age of the patients, illness duration, UPDRS-III, MMSE, and LEED is statistically non-significantly associated with FA alterations in patients with PD.

## Discussion

As far as we know, this is the first coordinate-based meta-analysis focusing on TBSS studies comparing FA in patients with PD to HCs. Our study identified lower FA mainly in the body of the CC and the left IFOF in patients with PD using the AES-SDM meta-analytical approach. Remarkably, these findings remained reproducible in jackknife sensitivity analysis and some subgroup meta-analysis, especially the body of the CC, suggesting robust disruption of the WM microstructure in those regions.

Using SDM software, Albrecht et al. (2019) identified the reduction of FA in the CC and the MCPs, and Suo et al. (2020) revealed a decrease in FA in supplementary regions including the left IFOF and the right inferior longitudinal fasciculus when merging both VBA and TBSS studies comparing FA in patients with PD to HCs. We reproduced the FA reduction of the CC and the left IFOF in patients with PD. However, some discrepancies emerged between our meta-analysis and Albrecht's findings (Albrecht et al., 2019). For instance, we failed to confirm a decrease in FA in the MCPs. There may be several reasons for such discrepancies. First, TBSS has a relative strength in the precise estimation of WM compared to the traditional VBA analysis (Smith et al., 2006), and we only included TBSS studies to avoid the inconsistency of results originating from methodological differences. Second, different samples with diverse clinical characteristics were included in our meta-analysis. The UPDRS-III score was lower in patients with PD in our sample (mean score: 26.84) than Albrecht's sample (mean score: 32.5), which might explain why we did not find WM lesions in the MCPs—a structure that mainly participates in motor coordination (Hasegawa et al., 2018).

The first cluster we identified decreased FA with a peak in the body of CC, which is consistent with many previous studies (Auning et al., 2014; Kamagata et al., 2014; Canu et al., 2015; Jiang et al., 2015; Lucas-Jiménez et al., 2016; Vervoort et al., 2016; Wang et al., 2016; Galantucci et al., 2017; Guimarães et al., 2018; Ji et al., 2019) comparing the WM diffusional properties of patients with PD to HCs. The CC plays a major role in communicating sensory, motor, and cognitive information between two cerebral hemispheres, and different parts of the CC participate in diverse cortical information communication revealed by DTI or functional MRI studies (Hofer and Frahm, 2006; Fabri et al., 2014; Courtemanche et al., 2018). Our study found decreased FA in the body and the genu of the CC, suggesting an impairment in the interhemispheric information transformation of the prefrontal lobe, motor, and supplementary motor areas (SMAs). The prefrontal cortex is a part of the limbic system and is related to higher cognitive function; apathy or reduced activation of the prefrontal lobe has been reported in patients with PD with cognitive impairment (Brück et al., 2004; Nagano-Saito et al., 2005; Trujillo et al., 2015; Gao et al., 2017). Damage to the prefrontal WM and the genu of the CC was revealed to be mainly associated with poorer intellectual or executive functions in patients with PD (Baggio et al., 2012; Hattori et al., 2012; Kamagata et al., 2013; Randver, 2018; Chondrogiorgi et al., 2019). The motor cortex generates motorial neural impulses, and functional alterations in this region are related to many motor signs in patients with PD (Burciu and Vaillancourt, 2018). The SMAs are associated with motor imagery (Iseki et al., 2008; La Fougère et al., 2010) and participates in the coupling of movement and posture during tasks (Viallet et al., 1992; Jacobs et al., 2009). Dysfunction or atrophy of the SMAs has been consistently reported in studies of patients with PD (Rosenberg-Katz et al., 2013; Herz et al., 2014; Kübel et al., 2018). In addition, transcranial magnetic stimulation (TMS) treatment (Shirota et al., 2013; Eggers et al., 2015) targeting the SMAs has been demonstrated to improve the motor function (e. g., UPSRS-III scores) of PD patients. In line with these findings, decreased FA in the body of the CC has been demonstrated to be related to poorer motor function in patients with PD (Galantucci et al., 2014), implicating the damaged interhemispheric connectivity of motor areas and SMAs in patients with PD.

However, our finding of decreased FA in the CC body in patients with PD relative to HCs was contradictory to the findings from a previous study by Wen et al. (2016) that reported increased FA in the CC. This discrepancy may be due to the disease stage of PD since, in Wen's study, these WM changes were mainly presented in the patients with PD with Hoehn & Yahr stage I, which may reflect neural compensations (Sanjari Moghaddam et al., 2020) for dopaminergic deficiency in the very early disease stage.

Furthermore, the higher percentage of male patients with PD included in our meta-analysis was associated with increased FA values in the body of the CC. It has been reported that women with PD have a higher mortality rate and faster disease progression (Bjornestad et al., 2016; Heinzel et al., 2018). Our results indicate that male patients are likely to present with a lower rate of WM degeneration. Additionally, men's WM accounted for a higher percentage of the whole-brain volume (Cosgrove et al., 2007) in a healthy population, which might contribute to this result as well.

In addition to the robust finding of decreased FA in the body of the CC, we also identified lower FA in the long fibers crossing the left temporal lobe, especially the left IFOF, in agreement with the results of previous studies (Rae et al., 2012; Gallagher et al., 2013; Theilmann et al., 2013; Lucas-Jiménez et al., 2016; Wang et al., 2016; Guimarães et al., 2018). The IFOF, a direct pathway connecting the occipital, posterior temporal, and orbitofrontal areas (Wu et al., 2016), plays an important role in visual-perceptual performance (Ashtari, 2012) and cognitive (Duncan et al., 2016) performance. Visual elaboration (Chen et al., 2013; Ekker et al., 2017) resulting from IFOF impairment might account for symptoms such as postural instability and gait difficulties (Wang et al., 2016; Tan et al., 2019) in patients with PD, and patients with predominant postural instability and gait difficulties were more likely to develop dementia in the late stage of disease (Lee et al., 2015). The current study included samples with the freezing of gait and samples with visual hallucinations, both of which have been associated with cognitive impairment in PD (Vercruysse et al., 2015; Firbank et al., 2018; Quattrone et al., 2019). The reduction of FA of WM located in the left temporal region in the present study is consistent with the lateral principle (Colosimo et al., 2010; Riederer et al., 2018) in patients with PD. Another meta-analysis (Xu et al., 2016) revealed that PD patients with mild cognitive impairment (PD-MCI) presented prominent gray matter volume (GMV) reduction in the left temporal-frontal regions, while PD patients with dementia (PDD) had bilateral reductions in those areas. The unilateral-to-bilateral development of GMV reduction in the frontal-limbic-temporal regions is a possible indicator of PD-MCI to PDD progression (Xu et al., 2016). Thus, the decreased FA in the left temporal lobe might underlie the early WM pathology of PD.

## Limitations

Although this meta-analysis avoids some methodological differences by only including TBSS studies and indicates possible stable WM microstructure alterations in PD, it still has some limitations. First, the differences in scanning parameters, field strength, and MR scanner vendors between studies were impossible to eliminate by statistical means. Second, FA is a dimensional value which cannot illustrate the axonal or myelin pathology of WM (Rae et al., 2012). It might be more helpful to combine other parameters (MD, AD, and RD) with FA in a future analysis. Third, publication bias could not be avoided, although we attempted to include as many studies as possible (even if they were negative). Finally, our study uses the coordinates from the included studies rather than their original *t*-statistic maps, which limits the accuracy of our results. Image-based meta-analysis should be considered as an improved alternative in future studies.

## Conclusion

The current quantitative meta-analysis of TBSS studies provides convergent evidence that WM disorganization exists in patients with PD and that the most robust WM abnormalities are in the body of the CC and the left IFOF, suggesting that abnormalities of interhemispheric connections and the disconnection of the left hemispheric cortical lobes might be the basis of the pathogenesis of PD. Future MRI studies could further investigate the diagnostic and prognostic value of WM microstructural abnormalities in patients with PD.

## Data Availability Statement

The original contributions generated for the study are included in the article/Supplementary Material, further inquiries can be directed to the corresponding author/s.

## Author Contributions

CL conceived the project and designed the protocol. XW obtained the data and analyzed the results. XW and CL wrote the main manuscript. CL and SL revised the manuscript. All authors critically reviewed the manuscript.

## Conflict of Interest

The authors declare that the research was conducted in the absence of any commercial or financial relationships that could be construed as a potential conflict of interest.
