# Supplementary material for: White Matter Abnormalities in Patients With Parkinson's Disease: A Meta-Analysis of Diffusion Tensor Imaging Using Tract-Based Spatial Statistics
Source: Front Aging Neurosci. 2021 Jan 28;12:610962. doi: 10.3389/fnagi.2020.610962 (PMC7876070; doi:10.3389/fnagi.2020.610962)
Supplement: Supplementary file 1 [file Table_1.docx]

**Supplementary Material**

Content of supplementary material

Supplementary Methods

Supplementary Results

Table S1. The quality assessment checklist and the scores of the included studies of FA.

Table S2. Jackknife sensitivity analysis of FA (* “Yes” indicates that the specific region of FA reduction was significant in the specific jackknife analysis; “No” indicates that the specific region of FA reduction was not significant in specific analysis.).

Table S3. Subgroup meta-analysis of studies in patients with PD compared with HC.

Table S4. Regional differences in FA between patients with PD and HC using FWHM 10mm in SDM.

Table S5. Demographic and clinical characteristics of participants in the 15 PD studies (19 data sets) included in the meta-analysis of MD.

Table S6. Regional differences in MD between patients with PD and HC.

Figure S1. Results of funnel plot analysis to test for publication bias of FA.

Figure S2. Flow chart for identifying PD studies of MD.

Figure S3. Results of funnel plot analysis to test for publication bias of MD.

Supplementary Methods

1.Selection criteria

**The inclusion criteria:**(i) original articles published in peer-reviewed English-language journals; (ii) studies comparing the FA values of WM areas between patients with PD and healthy controls; and (iii) studies that detected FA alterations in whole-brain analyses and reported the results in Talairach or Montreal Neurological Institute coordinates.

**The exclusion criteria** for this meta-analysis were as follows: (i) studies that were case reports or reviews; (ii) studies that lacked a healthy control group; and (iii) studies using overlapping research samples from different publications (in such cases, the data from the study with the largest sample were included in the meta-analysis).

## 2. Meta-analysis of abnormalities in fractional anisotropy

The AES-SDM technique uses effect sizes to combine reported peak coordinates that are extracted from databases with statistical parametric maps, and it recreates original maps of the effect size of FA difference in white matter between patients and controls. We performed the analysis as described in the AES-SDM tutorial and related publications(Radua and Mataix-Cols, 2009;Radua et al., 2014). The analytical parameters of the AES-SDM applied in the present study were as follows: voxel threshold p = 0.005, peak height threshold Z = 1.00, and cluster size threshold = 100 voxels, which has been used in previous studies(Li et al., 2012;Yang et al., 2019). We used MRIcron software (www.mricro.com/mricron/) to visualize AES-SDM maps overlaid on pooled analysis onto a high-resolution brain template generated by the International Consortium for Brain Mapping. When the sample size was sufficient, we conducted sensitivity subgroup analyses to test the robustness of the statistically significant findings by excluding studies with potential confounds on a one-off basis. We conducted five subgroup meta-analysis of studies: (i) studies with 3T scanner: 26 datasets including 819 patients (508 males, mean age 64.17yr, mean disease duration 5.74yr, mean UPDRS-III 27.77) with PD and 729 healthy controls; (ii) studies with corrected results: 28 datasets including 853 patients (males 512, mean age 64.81yr, mean disease duration 5.58yr, mean UPDRS-III27.52) with PD and 771 healthy controls; (iii) studies with b-value of 1000 s/mm2: 21 datasets including 718 patients (males 445, mean age 65.05yr, mean disease duration 5.56yr, mean UPDRS-III 27.56yr) with PD and 637 healthy controls. Cause coordinate based meta-analysis has inherent limitations related to the fixed FWHM used in the kernel, we did the main meta-analysis with FWHM of 10mm as supplementary.

3. Jackknife sensitivity, heterogeneity and publication bias analysis

To assess the robustness of the findings, we conducted a systematic whole-brain voxel-based jackknife analysis, in which we iteratively repeated the analysis, excluding 1 data set at a time to establish the extent to which the results could be replicated. If a brain region remained significant in all or most of the combinations of studies, we considered the finding to be highly replicable.

Analysis of heterogeneity and publication bias Heterogeneity refers to between-study variations. we examined the statistical (between-study) heterogeneity of individual clusters using a random-effects model with Q statistics, the parameters are the same as mentioned above. Moreover, we assessed publication bias by testing funnel plots using the Egger test(Egger et al., 1997) via STATA (www.stata.cn), in which any result showing p < 0.05 was regarded as having significant publication bias.

4. Meta-regression analysis

We performed meta-regression analyses using age, percentage of male patients, symptom severity (Unified Parkinson’s Disease Rating Scale motor score), illness duration in each study as the independent variables. The results were weighted by the square root of the sample size. To minimize the reporting of spurious relationships, we selected a more conservative threshold of p = 0.0005 as used in previous studies(Chen et al., 2016;Li et al., 2020), requiring abnormalities to be detected both in the slope and in one of the extremes of the regressor, and discarding findings in regions other than those detected in the main analyses.

5. Fiber tracking

We used DTIquery software (http://graphics.stanford.edu/ projects/dti/) and an atlas of human white-matter anatomy(Wakana et al., 2004) to help identify the most probable white-matter tracts passing through the clusters of voxels that showed significant FA group difference. The sample data of a healthy 35-year-old male provided by the DTIquery software was used. We mapped the white-matter tracts using streamline tracking techniques and filtered them by tract length and a box-shaped region of interest centered on the coordinates of significant clusters.

6. MD alteration in the patients with PD compared with HC

The following keywords were used to search pertinent articles published up to December 2020: “Parkinson” or “Parkinson’s disease”; “tract-based spatial statistical” or “diffusion tensor” or “mean diffusivity”. The flow diagram of literature search was presented in Figure S2. The inclusion and exclusion criteria were similar to FA literatures. The flow diagram of the literature search is presented in **Figure S2.**

Using SDM software, we explored the abnormalities of MD in patients with PD comparing to HC. Meanwhile, the jackknife sensitivity, heterogeneity and publication bias analysis carried out in the same way with FA analysis.

Supplementary Results

After systematic evaluation, 15 whole-brain TBSS studies relating to MD met our inclusion criteria. Four of them compared separate independent patient subgroups with the same healthy control groups. Thus, a total of 19 datasets including 846 patients (516 males) with PD and 778 HC (423 males) were included in the current analysis. The demographic and clinical characteristics of the PD studies included in the meta-analysis of MD were presented in **Table S5**. MD increasing was identified in the right lenticular nucleus (103 voxels; z = 1.214; P <0.001) **(Table S6)**, without statistically significant between-group heterogeneity or publication biases **(Figure S3)**. The discrepancy of findings regarding to MD and FA could be accounted by the difference in patient characteristic, e.g. right-dominant symptom PD patients showed MD increase in the included study (Pelizzari et al., 2020). Because the limited coordinates of MD reported in the whole brain TBSS study, MD increase in the right lenticular should be treated with caution.

Table S1. The quality assessment checklist and the scores of the included studies of FA.

| Score/study | (Pelizzari et al., 2020) | (Inguanzo et al., 2020) | (Quattrone et al., 2019) | (Guan et al., 2019) | (Li et al., 2018) | (Minett et al., 2018) | (Firbank et al., 2018) | (Luo et al., 2017) | (Chen et al., 2017) | (Georgiopoulos et al., 2017) | (Acosta-Cabronero et al., 2017) | (Vervoort et al., 2016) | (Wen et al., 2016) | (Vercruysse et al., 2015) | (Ji et al., 2015) | (Diez-Cirarda et al., 2015) | (Worker et al., 2014) | (Carriere et al., 2014) | (Agosta et al., 2014) | (Kamagata et al., 2013) | (Kim et al., 2013) | (Melzer et al., 2013) | (Hattori et al., 2012) |
| --- | --- | --- | --- | --- | --- | --- | --- | --- | --- | --- | --- | --- | --- | --- | --- | --- | --- | --- | --- | --- | --- | --- | --- |
| **Category 1: Participants** |  |  |  |  |  |  |  |  |  |  |  |  |  |  |  |  |  |  |  |  |  |  |  |
| 1 Patients were evaluated prospectively, specific diagnostic criteria were applied, and demographic data were reported | 1 | 1 | 1 | 1 | 1 | 1 | 1 | 1 | 1 | 1 | 1 | 1 | 1 | 1 | 1 | 1 | 1 | 1 | 1 | 1 | 1 | 1 | 1 |
| 2 Healthy comparison participants were evaluated prospectively; psychiatric and medical illnesses were excluded | 1 | 1 | 1 | 1 | 1 | 1 | 1 | 1 | 1 | 1 | 1 | 1 | 1 | 1 | 1 | 1 | 1 | 1 | 1 | 1 | 1 | 1 | 1 |
| 3 Important variables (e.g., age, gender, medication status, comorbidity, and subtype) were checked either via stratification or statistics | 1 | 1 | 1 | 1 | 1 | 1 | 1 | 1 | 1 | 1 | 1 | 1 | 1 | 1 | 1 | 1 | 1 | 1 | 1 | 1 | 1 | 1 | 1 |
| 4 All patients were comorbidity free | 1 | 1 | 1 | 1 | 1 | 1 | 1 | 1 | 1 | 1 | 1 | 1 | 1 | 1 | 1 | 1 | 1 | 1 | 1 | 1 | 1 | 1 | 1 |
| 5 All patients were medication naive | 0 | 0 | 0 | 0 | 0 | 1 | 0 | 0 | 0 | 0 | 0 | 0 | 1 | 0 | 0 | 0 | 0 | 0 | 0 | 0 | 0 | 0 | 0 |
| 6 Sample size per group: ≥ 20 scores 1, ≥ 10 scores 0.5 | 0 | 0.5 | 1 | 1 | 1 | 1 | 0.5 | 1 | 0.5 | 0.5 | 1 | 0.5 | 1 | 0.5 | 1 | 0.5 | 0.5 | 0.5 | 1 | 0.5 | 1 | 1 | 1 |
| **Category 2: Methods for image acquisition and analysis** |  |  |  |  |  |  |  |  |  |  |  |  |  |  |  |  |  |  |  |  |  |  |  |
| 7 Magnet strength: 3T scores 1, 1.5T scores 0.5 | 0.5 | 1 | 1 | 1 | 1 | 1 | 1 | 1 | 1 | 1 | 1 | 1 | 1 | 1 | 1 | 1 | 1 | 1 | 0.5 | 1 | 1 | 1 | 0.5 |
| 8 Number of diffusion gradient directions: ≥ 20 scores 1, ≥ 12 scores 0.5 | 1 | 1 | 1 | 0.5 | 1 | 1 | 1 | 1 | 1 | 1 | 1 | 1 | 1 | 1 | 1 | 1 | 1 | 0.5 | 0.5 | 1 | 0.5 | 1 | 0.5 |
| 9 b-value (s/mm2): ≥ 1,000 scores 1, < 1,000 scores 0.5 | 1 | 1 | 1 | 1 | 1 | 1 | 1 | 1 | 1 | 0.5 | 1 | 1 | 1 | 1 | 1 | 1 | 1 | 1 | 1 | 1 | 0.5 | 1 | 1 |
| 10 Technical factors such as magnet strength, number of diffusion gradient direction, b-value, voxel resolution etc. are clearly declared. | 1 | 1 | 1 | 1 | 1 | 1 | 1 | 1 | 1 | 1 | 1 | 1 | 1 | 1 | 1 | 1 | 1 | 1 | 1 | 1 | 1 | 1 | 1 |
| 11 Analyzing pipeline and measurements were clearly described so that could be reproduced. | 1 | 1 | 1 | 1 | 1 | 1 | 1 | 1 | 1 | 1 | 1 | 1 | 1 | 1 | 1 | 1 | 1 | 1 | 1 | 1 | 1 | 1 | 1 |
| 12 Whole brain analysis was automated without a previously defined region | 1 | 1 | 1 | 1 | 1 | 1 | 1 | 1 | 1 | 1 | 1 | 1 | 1 | 1 | 1 | 1 | 1 | 1 | 1 | 1 | 1 | 1 | 1 |
| 13 Coordinates of decreased or increased FA reported in a standard space | 1 | 1 | 1 | 1 | 1 | 1 | 1 | 1 | 1 | 1 | 1 | 1 | 1 | 1 | 1 | 1 | 1 | 1 | 1 | 1 | 1 | 1 | 1 |
| **Category 3: Results and conclusions** |  |  |  |  |  |  |  |  |  |  |  |  |  |  |  |  |  |  |  |  |  |  |  |
| 14 Statistical results were corrected for multiple comparison scores 1, uncorrected scores 0.5 | 1 | 1 | 1 | 1 | 1 | 1 | 1 | 1 | 1 | 1 | 1 | 1 | 1 | 1 | 1 | 1 | 1 | 1 | 1 | 1 | 1 | 1 | 1 |
| 15 Conclusions were consistent with the results obtained, and the limitations were discussed | 1 | 1 | 1 | 1 | 1 | 1 | 1 | 1 | 1 | 1 | 1 | 1 | 1 | 1 | 1 | 1 | 1 | 1 | 1 | 1 | 1 | 1 | 1 |
| Total score | 12.5 | 13.5 | 14 | 13.5 | 14 | 15 | 13.5 | 14 | 13.5 | 13 | 14 | 13.5 | 15 | 13.5 | 14 | 13.5 | 13.5 | 13 | 13 | 13.5 | 13 | 14 | 13 |
| Percent | 83% | 90% | 93% | 90% | 93% | 100% | 90% | 93% | 90% | 86% | 93% | 90% | 100% | 90% | 93% | 90% | 90% | 87% | 87% | 90% | 87% | 93% | 86% |

**Table S2. Jackknife sensitivity analysis of FA.**

| Discarded study | Corpus callosum | Left inferior network, inferior fronto-occipital fasciculus |
| --- | --- | --- |
| (Acosta-Cabronero et al., 2017) | Yes | Yes |
| (Agosta et al., 2014) | Yes | Yes |
| (Carriere et al., 2014)(Apathetic PD) | Yes | Yes |
| (Carriere et al., 2014)(Nonapathetic PD) | Yes | Yes |
| (Chen et al., 2017) | Yes | No |
| (Diez-Cirarda et al., 2015) | Yes | Yes |
| (Firbank et al., 2018)(PD-nonVH) | Yes | Yes |
| (Firbank et al., 2018)(PD-VH) | Yes | No |
| (Georgiopoulos et al., 2017) | Yes | Yes |
| (Guan et al., 2019) | Yes | Yes |
| (Hattori et al., 2012) | Yes | Yes |
| (Inguanzo et al., 2020;Pelizzari et al., 2020)(PD1) | Yes | Yes |
| (Inguanzo et al., 2020)(PD2) | Yes | Yes |
| (Inguanzo et al., 2020)(PD3) | Yes | Yes |
| (Ji et al., 2015) | Yes | Yes |
| (Kamagata et al., 2013) | Yes | Yes |
| (Kim et al., 2013) | Yes | Yes |
| (Li et al., 2018) | Yes | No |
| (Luo et al., 2017)(TD PD) | Yes | Yes |
| (Luo et al., 2017)(NoTD PD) | Yes | Yes |
| (Melzer et al., 2013) | Yes | Yes |
| (Minett et al., 2018) | Yes | Yes |
| (Pelizzari et al., 2020)(LPD) | Yes | Yes |
| (Pelizzari et al., 2020)(RPD) | Yes | Yes |
| (Quattrone et al., 2019) | Yes | Yes |
| (Vercruysse et al., 2015)(PD with FOG) | Yes | Yes |
| (Vercruysse et al., 2015)(PD without FOG) | Yes | Yes |
| (Vervoort et al., 2016)(TD PD) | Yes | Yes |
| (Wen et al., 2016) | Yes | Yes |
| (Worker et al., 2014) | Yes | Yes |
| Total | 30 out of 30 | 27out of 30 |

Abbreviations: PD = Parkinson Disease; RPD = right-dominant symptom PD patients; LPD = left-dominant symptom PD patients; PD1= PD1 had lower grey matter volumes than HC mainly in occipital and medial temporal; PD2 = PD2 had grey matter atrophy compared with HC mainly in bilateral orbital and prefrontal cortical regions; PD3 = PD3 did not show significant grey matter volume differences compared with HC; VH = visual hallucination; TD = tremor dominated; FOG=freezing of gait.

Table S3. Subgroup meta-analysis of studies in patients with PD compared with HC.

|  | WM Tract | Voxels | MNI Coordinates | | | SDM  Z score | p, uncorrected | Cluster breakdown  (voxels) |
| --- | --- | --- | --- | --- | --- | --- | --- | --- |
|  |  |  | X | Y | Z |  |  |  |
| Studies reported with corrected results | Corpus callosum | 278 | 12 | 14 | 24 | -1.919 | 0.000004888 | Corpus callosum (273) |
|  | Left inferior network, inferior fronto-occipital fasciculus | 109 | -42 | -18 | -12 | -1.281 | 0.000699103 | Left inferior network, inferior fronto-occipital fasciculus (43)  Corpus callosum (18)  Left optic radiations (12)  Left pons (12) |
| Studies with 3T scanner | Corpus callosum | 245 | 12 | 14 | 24 | -1.812 | ~0 | Corpus callosum (242) |
|  | Left inferior network, inferior fronto-occipital fasciculus | 118 | -42 | -18 | -12 | -1.244 | 0.000593603 | Left inferior network, inferior fronto-occipital fasciculus (44)  Corpus callosum (19)  Left cortico-spinal projections(12)  Left optic radiations (22)  Left pons (13)  Left inferior network, inferior longitudinal fasciculus (10) |
| Studies with b-value of 1000 s/mm2 | None |  |  |  |  |  |  |  |

* less than 10 voxels are not represented in the breakdown of voxels

Abbreviations: PD = Parkinson Disease; FA = fractional anisotropy; WM = white matter; MNI = Montreal Neurological Institute Space; SDM, Seed-based d Mapping; Jackknife: The jackknife sensitivity analysis column gives the number of studies whose omission does not affect the finding.

Table S4. Regional differences in FA between patients with PD and HC using FWHM 10mm in SDM.

| WM Tract | Voxels | MNI Coordinates | | | SDM  Z score | p, uncorrected | Cluster breakdown  (voxels) | Jackknife |
| --- | --- | --- | --- | --- | --- | --- | --- | --- |
|  |  | X | Y | Z |  |  |  |  |
| Corpus callosum | 210 | 12 | 14 | 24 | -1.572 | 0.000004888 | Corpus callosum (209) | 29/30 |

* less than 10 voxels are not represented in the breakdown of voxels

Abbreviations: PD = Parkinson Disease; FA = fractional anisotropy; WM = white matter; MNI = Montreal Neurological Institute Space; SDM, Seed-based d Mapping; Jackknife: The jackknife sensitivity analysis column gives the number of studies whose omission does not affect the finding.

Table S5. Demographic and clinical characteristics of participants in the 15 PD studies (19 data sets) included in the meta-analysis of MD.

| Study(subgroup) | PD | | | | | | | HC | | Study information | | | | | |
| --- | --- | --- | --- | --- | --- | --- | --- | --- | --- | --- | --- | --- | --- | --- | --- |
|  | participants(male) | age, yrs | Disease Duration | UPDRS-III | MMSE | Medication  status | LEDD, mg/day | participants(male) | age, yrs | Scanner | Direction | b value | Software | Threshold | major findings |
| (Pelizzari et al., 2020)(LPD) | 9(4) | 65.99 | 3.81* | 17 | NA | On-state | 158.85 | 17(9) | 63.16 | 1.5 | 64 | 1500 | FSL | P< 0.05 (FWE） | No significant MD alteration |
| (Pelizzari et al., 2020)(RPD) | 12(7) | 68.15 | 2.18* | 18.17 | NA | On-state | 269.06 | 17(9) | 63.16 | 1.5 | 64 | 1500 | FSL | P< 0.05 (FWE） | body of the CC, bilateral CI, right ACR, bilateral SCR, right PCR and SS, bilateral CE, bilateral SLF, right SFOF. |
| (Guan et al., 2019) | 65(32) | 55.5 | 4.7 | 27.1 | 27.8 | Off-state | NA | 46(21) | 57.8 | 3 | 15 | 1000 | FSL | p<0.001, cluster-based Corr | right forceps minor, left CE, Cingulum |
| (Firbank et al., 2018)(PD-nonVH) | 19(17) | 72.3 | NA | 34.7 | 25.6 | NA | 673.5 | 20(14) | 75.4 | 3 | 64 | 1000 | FSL | P< 0.05 (TFCE） | No significant MD alteration |
| (Luo et al., 2017)(TD PD) | 30(16) | 53.42 | 2 | 25.37 | NA | Off-state | 262 | 26(13) | 54.46 | 3 | 25 | 1000 | FSL | P< 0.05 (FWE） | the bilateral MCP, SCP, cerebral peduncles, thalamus, CI, and SCR, the fornix, inferior longitudinal fasciculi, and IFOF |
| (Luo et al., 2017)(NoTD PD) | 30(15) | 52.55 | 2.35 | 22.27 | NA | Off-state | 305 | 26(13) | 54.46 | 3 | 25 | 1000 | FSL | P< 0.05 (FWE） | No significant MD alteration |
| (Lee et al., 2017) | 21(11) | 66.2 | 7 | 16.4 | 28.2 | On-state | 805.2 | 30(11) | 68.6 | 3 | 15 | 800 | FSL | P< 0.05 (TFCE） | No significant MD alteration |
| (Acosta-Cabronero et al., 2017) | 25(20) | 63.6 | 6 | 16.3 | 26.7 | On-state | 748 | 50(28) | 63.6 | 3 | 30 | 1000 | FSL | P < 0.001, Uncorr | No significant MD alteration |
| (Vervoort et al., 2016)(PIGD) | 39(24) | 62.4 | 6.9 | 29.4 | 28.1 | Off-state | 525.7 | 19(14) | 58.1 | 3 | 61 | 1300 | FSL | P< 0.05 (TFCE） | No significant MD alteration |
| (Vervoort et al., 2016)(TD PD) | 16(9) | 55.1 | 4.8 | 28.9 | 28.9 | Off-state | 249.2 | 19(14) | 58.1 | 3 | 61 | 1300 | FSL | P< 0.05 (TFCE） | No significant MD alteration |
| (Wen et al., 2016) | 87(55) | 62.01 | 0.63 | 25.14 | NA | Drug-naïve | 0 | 60(40) | 60.33 | 3 | 64 | 1000 | FSL | P< 0.01 (TFCE） | No significant MD alteration |
| (Vercruysse et al., 2015)(PD with FOG) | 11(8) | 68.6 | 9.5 | 36.6 | 27.2 | On-state | 703.8 | 15(11) | 68.1 | 3 | 25/40/75 | 700/1000/2800 | FSL | P< 0.05 (FDR） | No significant MD alteration |
| (Vercruysse et al., 2015)(PD without FOG) | 15(11) | 67.6 | 7.6 | 32.5 | 28.3 | On-state | 461.3 | 15(11) | 68.1 | 3 | 25/40/75 | 700/1000/2800 | FSL | P< 0.05 (FDR） | No significant MD alteration |
| (Ji et al., 2015) | 20(11) | 64.2 | 4.64 | 28.76 | NA | Off-state | NA | 20(10) | 59.95 | 3 | 30 | 1000 | FSL | P<0.05 (TFCE_FWE） | No significant MD alteration |
| (Diez-Cirarda et al., 2015) | 37(22) | 67.97 | 6.96 | 21.72 | NA | On-state | 808.59 | 15(11) | 65.07 | 3 | 32 | 1000 | FSL | p<0.001, Uncorr | No significant MD alteration |
| (Worker et al., 2014) | 17(9) | 63.9 | 6.6 | 21.8 | 29.5 | On-state | NA | 17(9) | 63.9 | 3 | 64 | 1300 | FSL | P<0.017, TFCE &Bonferroni Corr | No significant MD alteration |
| (Agosta et al., 2014) | 43(29) | 65.8 | 9.1 | 32.6 | 27.6 | On-state | 607.1 | 33(17) | 64 | 1.5 | 12 | 1000 | FSL | P< 0.05 (FWE） | No significant MD alteration |
| (Kamagata et al., 2014) | 12(6) | 65.4 | 7.1 | NA | NA | On-state | 322.7 | 10(5) | 67.6 | 3 | 20 | 1000/2000 | FSL | P<0.017, TFCE &Bonferroni Corr | No significant MD alteration |
| (Kamagata et al., 2013) | 20(8) | 71.6 | 7.83 | NA | 25.7 | On-state | 464.2 | 20(10) | 72.7 | 3 | 32 | 1000 | FSL | P<0.05, (TFCE_FWE） | No significant MD alteration |
| Total | 846(516) | 64.64 | 7.73 |  | 27.00 |  | 508.59 | 778(423) | 64.61 |  |  |  |  |  |  |

Abbreviations: PD = Parkinson Disease; HC = healthy controls; UPDRS-III = Unified Parkinson Disease Rating Scale-III; RPD = right-dominant symptom PD patients; LPD = left-dominant symptom PD patients; TD = tremor dominated; FOG=freezing of gait; PIGD= postural instability and gait difficulty; H&Y = Hoehn &Yahr staging; NA = not available; MRI = Magnetic Resonance Image; Uncorr= uncorrected; Corr=corrected; FEW = family-wise error; TFCE = threshold-free cluster enhancement; MCP = middle cerebellar peduncle; UF = uncinate fasciculus; CC = Corpus callosum; CI = capsula interna; CE = capsula externa; ACR = anterior corona radiata; SCR = superior corona radiata; PCR = posterior corona radiata; SLF = superior longitudinal fasciculus; SFOF = superior fronto-occipital fasciculus

Table S6. Regional differences in MD between patients with PD and HC.

| WM Tract | Voxels | MNI Coordinates | | | SDM  Z score | P, uncorrected | Cluster breakdown (voxels) | Jackknife |
| --- | --- | --- | --- | --- | --- | --- | --- | --- |
|  |  | X | Y | Z |  |  |  |  |
| Right lenticular nucleus, putamen, BA 48 | 103 | 28 | 14 | -8 | 1.214 | 0.000068665 | Right inferior network, inferior longitudinal fasciculus(23)  Right inferior network, inferior fronto-occipital fasciculus(20)  Right lenticular nucleus, putamen, BA 48(20)  (undefined), BA 48(31) | 16/19 |

* less than 10 voxels are not represented in the breakdown of voxels

Abbreviations: PD = Parkinson Disease; FA = fractional anisotropy; WM = white matter; MNI = Montreal Neurological Institute Space; SDM, Seed-based d Mapping; Jackknife: The jackknife sensitivity analysis column gives the number of studies whose omission does not affect the finding.

Figure S1. Results of funnel plot analysis to test for publication bias of FA.
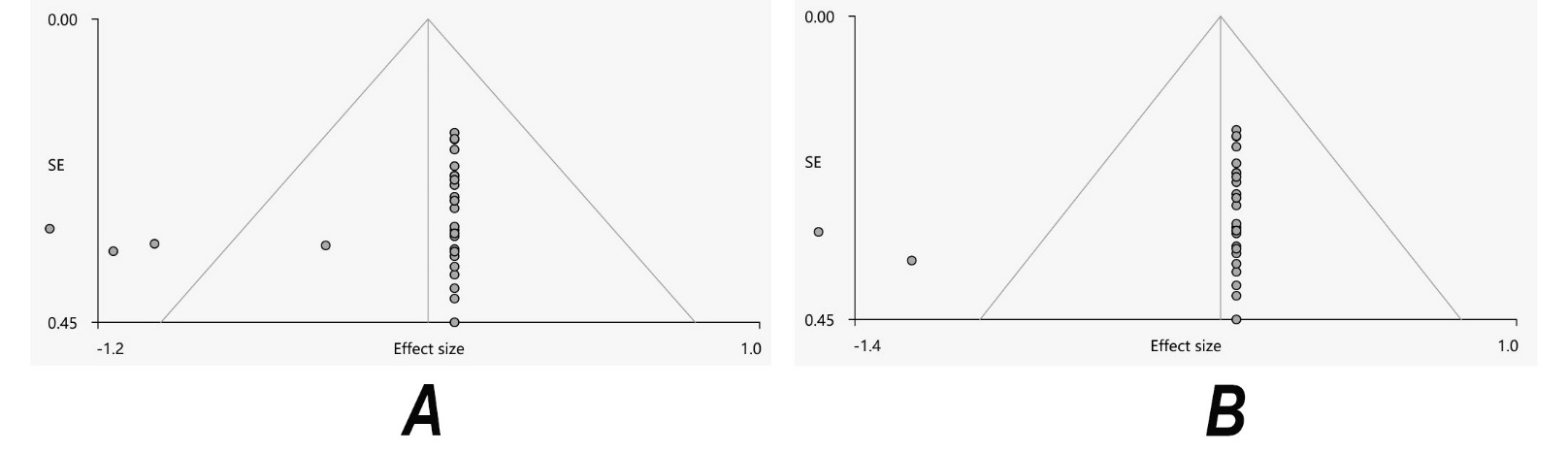


For the current pooled meta-analysis, the Egger’s test and funnel plots revealed no significant publication bias (A) in the CC (Z=-1.10, t=-1.49, df=28, P= 0.148), (B) in the left IFOF (Z=-0.83, t=-1.17, df=28, P=0.252) in patients with PD than controls. Abbreviations: CC = corpus callosum; IFOF = inferior fronto-occipital fasciculus; PD = Parkinson Disease; SE = standard error.

Figure S2. Flow chart for identifying PD studies of MD.


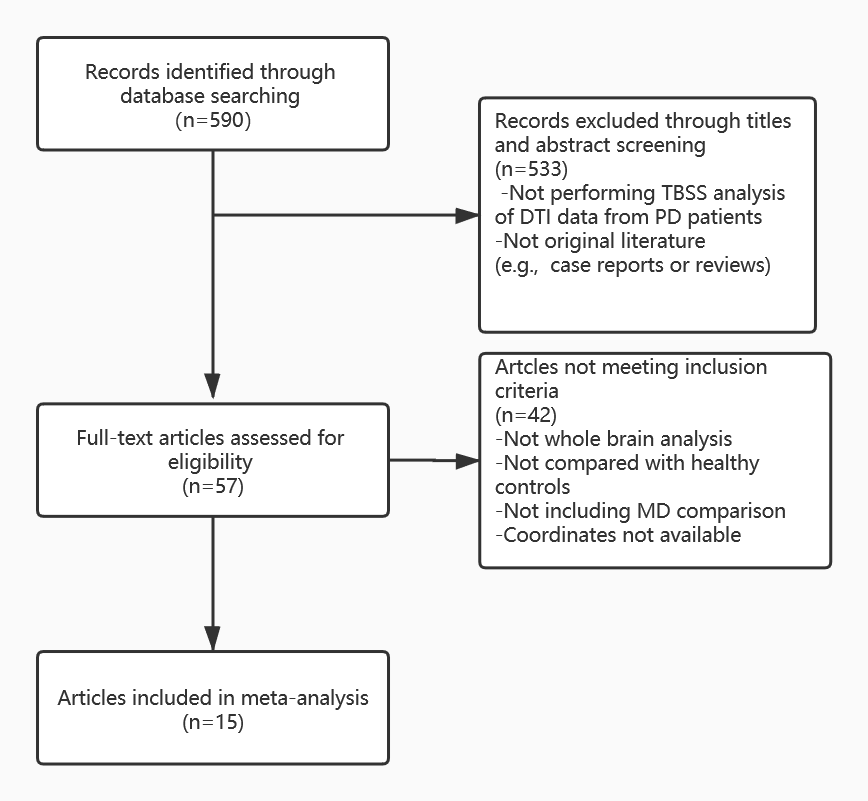


Figure S3. Results of funnel plot analysis to test for publication bias of MD.


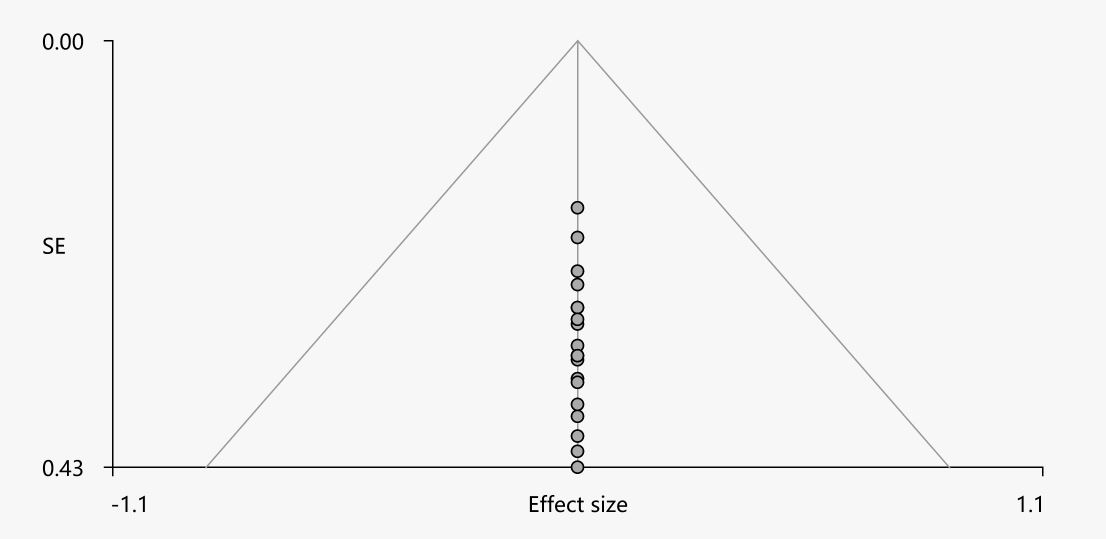


For the current pooled meta-analysis, the Egger’s test and funnel plots revealed no significant publication bias in the right lenticular nucleus, putamen; PD = Parkinson Disease; SE = standard error.

**References**

Acosta-Cabronero, J., Cardenas-Blanco, A., Betts, M.J., Butryn, M., Valdes-Herrera, J.P., Galazky, I., et al. (2017). The whole-brain pattern of magnetic susceptibility perturbations in Parkinson's disease. *Brain* 140**,** 118-131.doi:10.1093/brain/aww278

Agosta, F., Canu, E., Stefanova, E., Sarro, L., Tomić, A., Špica, V., et al. (2014). Mild cognitive impairment in Parkinson's disease is associated with a distributed pattern of brain white matter damage. *Human Brain Mapping* 35**,** 1921-1929.doi:10.1002/hbm.22302

Carriere, N., Besson, P., Dujardin, K., Duhamel, A., Defebvre, L., Delmaire, C., et al. (2014). Apathy in Parkinson's disease is associated with nucleus accumbens atrophy: A magnetic resonance imaging shape analysis. *Movement Disorders* 29**,** 897-903.doi:10.1002/mds.25904

Chen, B., Fan, G., Sun, W., Shang, X., Shi, S., Wang, S., et al. (2017). Usefulness of diffusion-tensor MRI in the diagnosis of Parkinson variant of multiple system atrophy and Parkinson's disease: a valuable tool to differentiate between them? *Clinical Radiology* 72**,** 610.e619-610.e615.doi:10.1016/j.crad.2017.02.005

Chen, L., Hu, X., Ouyang, L., He, N., Liao, Y., Liu, Q., et al. (2016). A systematic review and meta-analysis of tract-based spatial statistics studies regarding attention-deficit/hyperactivity disorder. *Neurosci Biobehav Rev* 68**,** 838-847.doi:10.1016/j.neubiorev.2016.07.022

Diez-Cirarda, M., Ojeda, N., Pena, J., Cabrera-Zubizarreta, A., Gomez-Beldarrain, M.A., Gomez-Esteban, J.C., et al. (2015). Neuroanatomical Correlates of Theory of Mind Deficit in Parkinson's Disease: A Multimodal Imaging Study. *PLoS One* 10**,** e0142234.doi:10.1371/journal.pone.0142234

Egger, M., Smith, G.D., and Phillips, A.N. (1997). Meta-analysis: principles and procedures. *BMJ (Clinical research ed.)* 315**,** 1533-1537.doi:10.1136/bmj.315.7121.1533

Firbank, M.J., Parikh, J., Murphy, N., Killen, A., Allan, C.L., Collerton, D., et al. (2018). Reduced occipital GABA in Parkinson disease with visual hallucinations. *Neurology* 91**,** e675-e685.doi:10.1212/WNL.0000000000006007

Georgiopoulos, C., Warntjes, M., N, D.I., Zachrisson, H., Engström, M., Haller, S., et al. (2017). Olfactory impairment in Parkinson's disease studied with diffusion tensor and magnetization transfer imaging. *Journal of Parkinson's Disease* 7**,** 301-311.doi:10.3233/JPD-161060

Guan, X., Huang, P., Zeng, Q., Liu, C., Wei, H., Xuan, M., et al. (2019). Quantitative susceptibility mapping as a biomarker for evaluating white matter alterations in Parkinson’s disease. *Brain Imaging and Behavior* 13**,** 220-231.doi:10.1007/s11682-018-9842-z

Hattori, T., Orimo, S., Aoki, S., Ito, K., Abe, O., Amano, A., et al. (2012). Cognitive status correlates with white matter alteration in Parkinson's disease. *Human Brain Mapping* 33**,** 727-739.doi:10.1002/hbm.21245

Inguanzo, A., Sala-Llonch, R., Segura, B., Erostarbe, H., Abos, A., Campabadal, A., et al. (2020). Hierarchical cluster analysis of multimodal imaging data identifies brain atrophy and cognitive patterns in Parkinson's disease. *Parkinsonism Relat Disord* 82**,** 16-23.doi:10.1016/j.parkreldis.2020.11.010

Ji, L., Wang, Y., Zhu, D., Liu, W., and Shi, J. (2015). White matter differences between multiple system atrophy (parkinsonian type) and parkinson's disease: A diffusion tensor image study. *Neuroscience* 305**,** 109-116.doi:10.1016/j.neuroscience.2015.07.060

Kamagata, K., Motoi, Y., Tomiyama, H., Abe, O., Ito, K., Shimoji, K., et al. (2013). Relationship between cognitive impairment and white-matter alteration in Parkinson's disease with dementia: Tract-based spatial statistics and tract-specific analysis. *European Radiology* 23**,** 1946-1955.doi:10.1007/s00330-013-2775-4

Kamagata, K., Tomiyama, H., Hatano, T., Motoi, Y., Abe, O., Shimoji, K., et al. (2014). A preliminary diffusional kurtosis imaging study of Parkinson disease: Comparison with conventional diffusion tensor imaging. *Neuroradiology* 56**,** 251-258.doi:10.1007/s00234-014-1327-1

Kim, H.J., Kim, S.J., Kim, H.S., Choi, C.G., Kim, N., Han, S., et al. (2013). Alterations of mean diffusivity in brain white matter and deep gray matter in Parkinson's disease. *Neuroscience Letters* 550**,** 64-68.doi:10.1016/j.neulet.2013.06.050

Lee, W.W., Yoon, E.J., Lee, J.Y., Park, S.W., and Kim, Y.K. (2017). Visual Hallucination and Pattern of Brain Degeneration in Parkinson's Disease. *Neurodegenerative Diseases* 17**,** 63-72.doi:10.1159/000448517

Li, J., Pan, P., Huang, R., and Shang, H. (2012). A meta-analysis of voxel-based morphometry studies of white matter volume alterations in Alzheimer's disease. *Neurosci Biobehav Rev* 36**,** 757-763.doi:10.1016/j.neubiorev.2011.12.001

Li, Q., Zhao, Y., Chen, Z., Long, J., Dai, J., Huang, X., et al. (2020). Meta-analysis of cortical thickness abnormalities in medication-free patients with major depressive disorder. *Neuropsychopharmacology* 45**,** 703-712.doi:10.1038/s41386-019-0563-9

Li, X.R., Ren, Y.D., Cao, B., and Huang, X.L. (2018). Analysis of white matter characteristics with tract-based spatial statistics according to diffusion tensor imaging in early Parkinson's disease. *Neuroscience Letters* 675**,** 127-132.doi:10.1016/j.neulet.2017.11.064

Luo, C.Y., Song, W., Chen, Q., Yang, J., Gong, Q.Y., and Shang, H.F. (2017). White matter microstructure damage in tremor-dominant Parkinson’s disease patients. *Neuroradiology* 59**,** 691-698.doi:10.1007/s00234-017-1846-7

Melzer, T.R., Watts, R., Macaskill, M.R., Pitcher, T.L., Livingston, L., Keenan, R.J., et al. (2013). White matter microstructure deteriorates across cognitive stages in Parkinson disease. *Neurology* 80**,** 1841-1849.doi:10.1212/WNL.0b013e3182929f62

Minett, T., Su, L., Mak, E., Williams, G., Firbank, M., Lawson, R.A., et al. (2018). Longitudinal diffusion tensor imaging changes in early Parkinson’s disease: ICICLE-PD study. *Journal of Neurology* 265**,** 1528-1539.doi:10.1007/s00415-018-8873-0

Pelizzari, L., Di Tella, S., Laganà, M.M., Bergsland, N., Rossetto, F., Nemni, R., et al. (2020). White matter alterations in early Parkinson's disease: role of motor symptom lateralization. *Neurol Sci* 41**,** 357-364.doi:10.1007/s10072-019-04084-y

Quattrone, A., Caligiuri, M.E., Morelli, M., Nigro, S., Vescio, B., Arabia, G., et al. (2019). Imaging counterpart of postural instability and vertical ocular dysfunction in patients with PSP: A multimodal MRI study. *Parkinsonism and Related Disorders*.doi:10.1016/j.parkreldis.2019.02.022

Radua, J., Grau, M., Van Den Heuvel, O.A., Thiebaut De Schotten, M., Stein, D.J., Canales-Rodriguez, E.J., et al. (2014). Multimodal voxel-based meta-analysis of white matter abnormalities in obsessive-compulsive disorder. *Neuropsychopharmacology* 39**,** 1547-1557.doi:10.1038/npp.2014.5

Radua, J., and Mataix-Cols, D. (2009). Voxel-wise meta-analysis of grey matter changes in obsessive-compulsive disorder. *Br J Psychiatry* 195**,** 393-402.doi:10.1192/bjp.bp.108.055046

Vercruysse, S., Leunissen, I., Vervoort, G., Vandenberghe, W., Swinnen, S., and Nieuwboer, A. (2015). Microstructural changes in white matter associated with freezing of gait in Parkinson's disease. *Movement Disorders* 30**,** 567-576.doi:10.1002/mds.26130

Vervoort, G., Leunissen, I., Firbank, M., Heremans, E., Nackaerts, E., Vandenberghe, W., et al. (2016). Structural brain alterations in motor subtypes of Parkinson's disease: Evidence from probabilistic tractography and shape analysis. *PLoS ONE* 11.doi:10.1371/journal.pone.0157743

Wakana, S., Jiang, H., Nagae-Poetscher, L.M., Van Zijl, P.C.M., and Mori, S. (2004). Fiber tract-based atlas of human white matter anatomy. *Radiology* 230**,** 77-87.doi:10.1148/radiol.2301021640

Wen, M.C., Heng, H.S., Ng, S.Y., Tan, L.C., Chan, L.L., and Tan, E.K. (2016). White matter microstructural characteristics in newly diagnosed Parkinson's disease: An unbiased whole-brain study. *Scientific reports* 6**,** 35601.doi:10.1038/srep35601

Worker, A., Blain, C., Jarosz, J., Chaudhuri, K.R., Barker, G.J., Williams, S.C.R., et al. (2014). Diffusion tensor imaging of Parkinson's disease, multiple system atrophy and progressive supranuclear palsy: A tract-based spatial statistics study. *PLoS ONE* 9.doi:10.1371/journal.pone.0112638

Yang, C., Li, L., Hu, X., Luo, Q., Kuang, W., Lui, S., et al. (2019). Psychoradiologic abnormalities of white matter in patients with bipolar disorder: diffusion tensor imaging studies using tract-based spatial statistics. *J Psychiatry Neurosci* 44**,** 32-44.doi:10.1503/jpn.170221
